# Precision medicine for futile recanalization after mechanical thrombectomy in acute ischemic stroke: mechanisms, diagnostics, and tailored therapies

**DOI:** 10.3389/fneur.2026.1962902

**Published:** 2026-10-08

**Authors:** Yihua Zhu, Jie Wen, Xingqi Zhao, Haoxuan Li, Gelin Xu

**Affiliations:** 1 Guangxi University of Chinese Medicine, Nanning, China; 2 Department of Neurology, Shenzhen Second People's Hospital, The First Affiliated Hospital of Shenzhen University, Shenzhen, China; 3 School of Medicine, Shenzhen University, Shenzhen, China

**Keywords:** artificial intelligence, biomarkers, cerebrovascular disease, endovascular thrombectomy, futile recanalization, ischemic stroke, personalized medicine, precision medicine

## Abstract

Mechanical thrombectomy has substantially improved outcomes in appropriately selected patients with acute ischemic stroke, yet futile recanalization remains a major clinical challenge. Futile recanalization describes poor functional recovery despite technically successful macrovascular reperfusion, underscoring the incomplete relationship between angiographic reperfusion and tissue-level or microvascular perfusion. This narrative review summarizes current clinical, mechanistic, and translational evidence on the determinants of futile recanalization and evaluates emerging precision-medicine approaches to its prevention and management. Precision imaging has established a role in tissue-based patient selection, and recent randomized trials have expanded thrombectomy evidence to selected patients with large-core infarction. In contrast, current randomized evidence does not support routine endovascular treatment for unselected medium-vessel occlusion populations. We further examine bridging therapy versus direct mechanical thrombectomy, individualized periprocedural management, biomarkers, and decision support based on artificial intelligence. Clinical and imaging phenotypes may provide prognostic or mechanistic information, but no phenotype-based strategy has been prospectively validated to select bridging therapy or direct thrombectomy. Biomarkers currently remain primarily prognostic or exploratory. Artificial intelligence applications are largely focused on diagnostic and prognostic support. treatment-benefit prediction, by contrast, requires evidence of treatment-effect modification and prospective validation. Experimental studies of microvascular no-reflow and thrombo-inflammation provide biological rationale for targeted interventions but do not establish clinical efficacy. Future research should integrate multimodal imaging, biological profiling, and validated decision-support tools to move beyond angiographic success toward individualized strategies focused on tissue integrity and meaningful functional recovery.

## 1 Introduction

Over the past decade, the therapeutic landscape of acute ischemic stroke (AIS) has been reshaped by mechanical thrombectomy (MT), a cornerstone of modern cerebrovascular disease management. Since the publication of five landmark randomized controlled trials (RCTs) in 2015, MT has been firmly established as the standard of care for patients with anterior circulation large vessel occlusion (LVO) (1, 2). Subsequent trials utilizing advanced multimodal neuroimaging provided evidence supporting the extension of the therapeutic window up to 24 h, substantially expanding the eligible population (3, 4). At present, international guidelines recommend MT for appropriately selected patients, and its application continues to broaden into highly specific clinical scenarios (5).

Despite high rates of successful reperfusion after MT, poor functional recovery remains common, highlighting the limitations of vessel-oriented endpoints. Although macrovascular recanalization is achieved in the majority of treated patients, up to 50% may fail to achieve functional independence despite successful reperfusion (6–8). Futile recanalization describes poor functional recovery despite technically successful macrovascular reperfusion, but its operational definition varies according to the reperfusion threshold, mTICI/eTICI grade, functional outcome cutoff, assessment time point, and baseline or pre-stroke disability (9). A commonly used definition combines TICI 2b–3 reperfusion with an mRS of 3–6 at 90 days, although mRS 3–6 encompasses heterogeneous degrees of disability; some studies therefore use mRS 4–6 as a more stringent cutoff. These thresholds are not interchangeable and may contribute to variation in reported rates (9). Importantly, macrovascular recanalization denotes reopening of the occluded artery, whereas TICI/eTICI grades reflect downstream angiographic perfusion; neither necessarily indicates adequate tissue-level or microvascular perfusion. Accordingly, this review preferentially uses eTICI, retaining mTICI for historical studies. More complete reperfusion (eTICI 2c/3) should not be considered biologically equivalent to eTICI 2b. In patients with pre-stroke disability, absolute post-stroke mRS thresholds may also incompletely capture meaningful recovery, making return toward pre-stroke function informative in selected patients (10). Although “futile reperfusion” more directly captures the disconnect between angiographic reperfusion and tissue recovery, “futile recanalization” is retained because of its established use in the literature and consistency with this review.

Current evidence suggests that futile recanalization is not driven by a single mechanism but is a multifactorial consequence of individual biological variability. Proposed contributing factors include specific infarct core topographies, impaired microvascular perfusion (no-reflow phenomenon), individualized inflammatory and immune responses to ischemia–reperfusion injury, and procedure-related complications (11–13). In the post-thrombectomy era, addressing this paradox requires an evolution in clinical philosophy. Successful recovery may depends not merely on macroscopic reperfusion but on a highly individualized interplay of collateral circulation, microvascular integrity, and tailored periprocedural physiology (14, 15).

Aligning with the global transition toward personalized medicine in cerebrovascular disease, this review focuses on how biological, procedural, and systemic factors contribute to futile recanalization. By critically examining precision diagnostics, tailored reperfusion strategies, and individualized periprocedural care, we aim to outline a paradigm shift from a uniform, recanalization-centric approach to a personalized, functional-recovery-oriented model (Figure 1).

**Figure 1:**
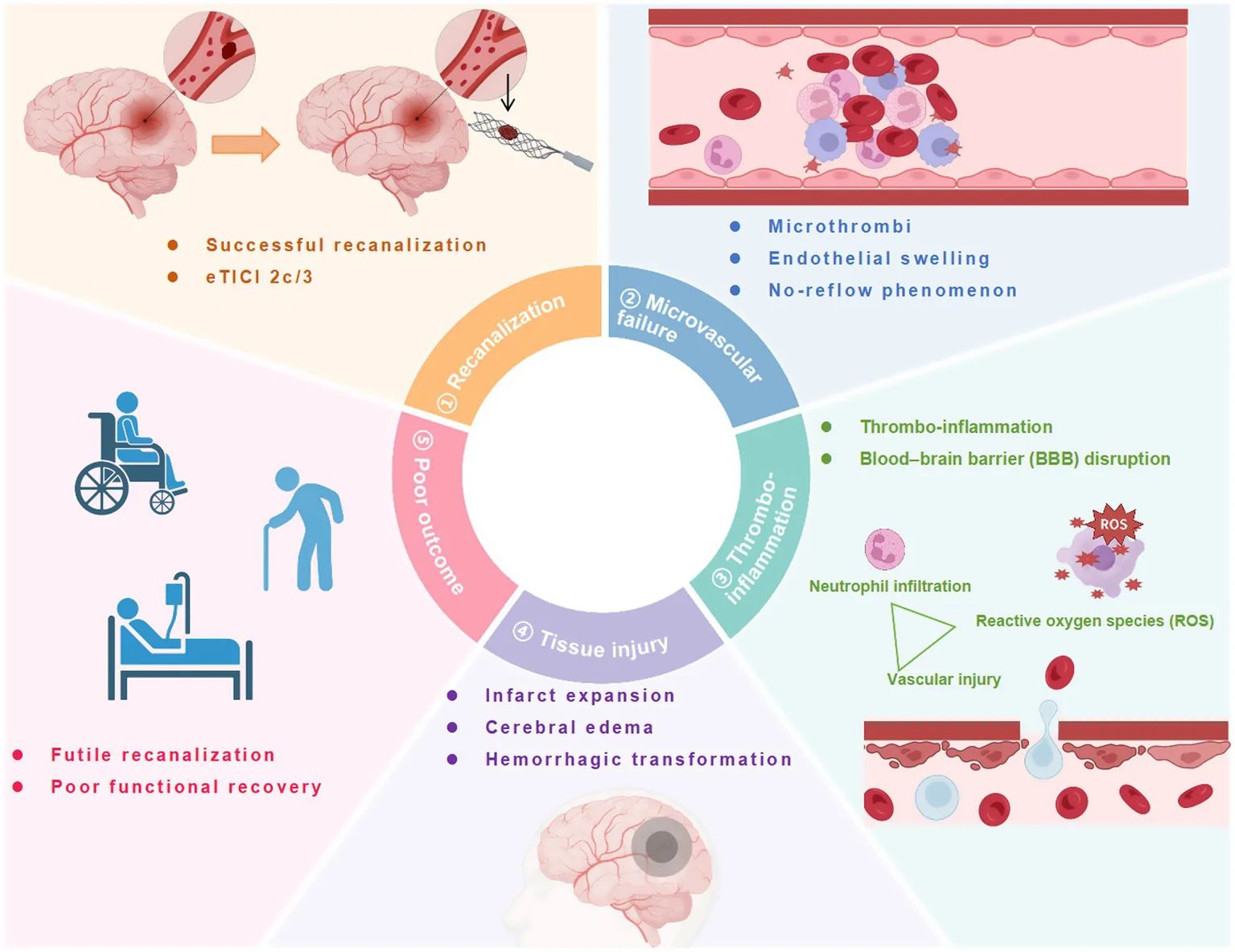
Multilevel pathophysiological cascade underlying futile recanalization after mechanical thrombectomy. This schematic integrates clinical and preclinical evidence on mechanisms that may contribute to futile recanalization. Following successful large-vessel recanalization, persistent impairment of downstream tissue perfusion may involve microvascular obstruction and reperfusion-associated thrombo-inflammatory responses, including microthrombi, endothelial swelling, no-reflow, neutrophil recruitment, reactive oxygen species (ROS) generation, and blood–brain barrier (BBB) disruption. These processes may contribute to subsequent tissue injury and poor functional recovery despite angiographic reperfusion, highlighting the multilevel pathophysiology underlying the clinical dissociation between recanalization and functional outcome. In this schematic, “no-reflow” refers to mechanistic microvascular failure, not to a direct imaging diagnosis. Created in BioRender. Zhu, Y. (2026) https://BioRender.com/w8zxety.

## 2 Literature search and selection principles

This narrative review was informed by a structured literature search of PubMed, Web of Science, and Embase from database inception to 12 Sep 2026. Searches were restricted to English-language, peer-reviewed publications. A core search was performed combining terms for acute ischemic stroke, MT, and futile recanalization. This was supplemented by additional searches using topic-specific terms addressing mechanistic and microcirculatory processes, reperfusion strategies, periprocedural management, precision diagnostics, biomarkers, imaging, and artificial intelligence.

We considered peer-reviewed clinical and preclinical studies, RCTs, systematic reviews and meta-analyses, formal clinical guidelines or consensus documents, and large observational studies addressing acute ischemic stroke in the context of endovascular therapy or MT. RCTs, meta-analyses, and contemporary guidelines were prioritized for clinical interpretation, whereas preclinical studies were selectively included to provide mechanistic context where human evidence was limited and were interpreted as hypothesis-generating rather than as evidence of clinical efficacy. Case reports, small case series (<10 patients), studies of non-ischemic cerebrovascular diseases, and publications lacking clinically or mechanistically relevant outcomes were excluded. Potentially overlapping cohorts or trials were assessed for the degree of overlap, with the most comprehensive or directly relevant report prioritized when analyses substantially overlapped. Given heterogeneity in study populations, reperfusion definitions, outcome measures, imaging characteristics, and study designs, the evidence was synthesized narratively. Details of the search strategy are provided in Supplementary Table 1.

## 3 The recanalization paradox: why successful reperfusion fails

### 3.1 Beyond eTICI: the limitations of macroscopic recanalization

The TICI framework has long served as a standard angiographic approach for assessing the extent of reperfusion after endovascular treatment. As defined previously, arterial or macrovascular recanalization denotes reopening of an occluded artery, whereas angiographic reperfusion assessed by eTICI reflects the extent of downstream angiographic perfusion after arterial reopening. In contemporary conceptual descriptions, eTICI is used to characterize different degrees of angiographic reperfusion, and these grades should not be treated as biologically equivalent. In particular, eTICI 2b, 2c, and 3 represent progressively more complete degrees of angiographic reperfusion, with higher grades (eTICI 2c/3) associated with better functional outcomes than eTICI 2b (16, 17). Nevertheless, angiographic reperfusion does not necessarily ensure adequate tissue-level or microvascular perfusion, and even patients achieving eTICI 3 may experience poor functional recovery (9, 11). Futile recanalization should not be interpreted as a purely angiographic failure or as synonymous with microvascular no-reflow; rather, it describes the clinical outcome paradox in which technically successful macrovascular reperfusion is not followed by satisfactory functional recovery. When historical studies used mTICI, their original terminology is retained rather than retrospectively relabeled.

Clinical variables such as advanced age, baseline hyperglycemia, and poor premorbid status have been associated with futile recanalization (18). Collateral circulation may also modify the relationship between arterial reperfusion and tissue outcome. Favorable leptomeningeal collaterals are associated with smaller ischemic core volumes and more favorable outcomes after endovascular treatment (19, 20). These clinical observations support a shift from an exclusively macroscopic assessment of reperfusion toward consideration of collateral status and downstream tissue-level determinants, while the relative contribution of individual microvascular mechanisms remains incompletely established.

### 3.2 Microvascular failure as a target for precision medicine

The dissociation between large-vessel recanalization and clinical outcome may involve impaired downstream tissue perfusion, including microvascular dysfunction or “no-reflow.” Clinical studies using postprocedural CT or MR perfusion imaging have identified persistent tissue-level hypoperfusion in a proportion of patients despite successful proximal arterial reopening (6, 21). Imaging-defined hypoperfusion encompasses several potentially overlapping processes, including incomplete macrovascular reperfusion, distal embolization, early reocclusion, inadequate collateral circulation, infarct progression, and microvascular dysfunction (20, 22). Persistent hypoperfusion should be interpreted as a marker of impaired downstream perfusion rather than as a direct measure of capillary no-reflow (23).

Preclinical and experimental studies have identified several mechanisms that may impair microvascular reperfusion after ischemia. These include pericyte responses, endothelial cell swelling, and leukocyte or platelet accumulation within the microcirculation, which may contribute to microvascular obstruction and impaired capillary reperfusion despite restoration of proximal arterial flow (24). Together, these mechanisms provide a biological basis for persistent tissue-level perfusion deficits after successful large-vessel recanalization.

Thrombo-inflammation represents another important component of post-reperfusion microvascular dysfunction. Preclinical evidence indicates that peripheral neutrophils may infiltrate the brain parenchyma after reperfusion, releasing pro-inflammatory mediators and forming neutrophil extracellular traps (NETs). These structures may promote microthrombus formation and endothelial injury (22, 25). The variability of these inflammatory responses may provide a rationale for investigating phenotype-informed adjunctive anti-inflammatory strategies (26).

Experimental and translational evidence further implicates endothelial injury in post-ischemic microvascular dysfunction. Mechanical manipulation during thrombectomy can produce endothelial injury, while pre-existing vascular pathology may influence individual susceptibility (27). Endothelial disruption may expose thrombogenic surfaces and contribute to secondary microthrombus formation. Reperfusion-related oxidative stress and reactive oxygen species (ROS) have also been implicated in endothelial and blood–brain barrier (BBB) injury (28, 29). These mechanisms provide potential biological targets for precision-oriented approaches to microcirculatory protection, although their translation into patient-specific therapeutic strategies remains an area of ongoing investigation. The evidence hierarchy and clinical readiness of key precision-medicine approaches relevant to futile recanalization are summarized in Table 1.

**Table 1:** Evidence hierarchy and clinical readiness of precision-medicine approaches for futile recanalization.

Phenotype/biomarker/strategy	Potential clinical decision	Evidence level	Principal limitation	Current clinical readiness
eTICI/angiographic reperfusion	Assessment of macrovascular reperfusion	Tier 1	Does not fully capture tissue-level perfusion or functional recovery	Established clinical practice
Tissue-window imaging/infarct core	MT eligibility and risk–benefit assessment in selected populations	Tier 1 for trial-supported selection	Trial-specific criteria; imaging is an imperfect surrogate of tissue biology	Established in selected clinical settings
Collateral circulation	Prognostic assessment and characterization of tissue viability	Tier 2	Prognostic association does not establish treatment-effect modification	Clinically available; not independently treatment-guiding
Frailty/pre-stroke functional status	Prognostic and individualized risk–benefit assessment	Tier 2	Strong prognostic value does not establish standalone treatment selection	Clinically informative; treatment-selection role not established
HIR/postprocedural perfusion measures	Prognostic stratification for secondary injury and functional outcome	Tier 2	Definitions/cutoffs vary; treatment benefit modification not established	Emerging prognostic application
Eosinophil count and other blood biomarkers	Risk stratification for secondary injury	Tier 2	Mainly observational evidence; limited validation for treatment selection	Exploratory
Pericyte, endothelial, NET, ROS and related mechanisms	Identification of potential biological targets	Tier 3	Predominantly experimental/translational evidence	Preclinical/translational
AI-based multimodal prediction	Diagnostic and prognostic decision support	Tier 4	External validation, calibration, dataset shift, bias, and prospective clinical impact remain concerns	Investigational
Targeted immunomodulation/multi-omics/nanotherapy	Future treatment stratification or adjunctive therapy	Tier 4	Limited clinical validation and uncertain treatment benefit	Investigational

HIR, hypoperfusion intensity ratio; MT, mechanical thrombectomy; NETs, neutrophil extracellular traps; ROS, reactive oxygen species. Tier 1: Established clinical evidence—supported by randomized trials, guidelines, or validated clinical practice. Tier 2: Clinical prognostic evidence—associations derived from observational studies, imaging, or biomarkers, not yet shown to modify treatment effects. Tier 3: Mechanistic/translational evidence—experimental or preclinical findings providing biological plausibility. Tier 4: Emerging therapeutic concepts—investigational approaches requiring prospective validation. Evidence levels reflect the current literature and may evolve as new data emerge.

## 4 Precision diagnostics: from time-based selection to tissue-based selection

### 4.1 Expanding thrombectomy eligibility through precision imaging

Historically, the “time is brain” paradigm dictated uniform treatment protocols, restricting MT to a rigid 6-h window. However, the DAWN and DEFUSE-3 trials catalyzed a shift toward tissue-based selection by leveraging advanced neuroimaging, including CT perfusion or multimodal MRI, to define tissue-based patient selection beyond conventional time thresholds (30, 31). By identifying selected patients with potentially salvageable tissue beyond conventional time thresholds, these trials supported expansion of MT indications up to 24 h (32). This transition from a predominantly time-based approach toward an imaging-informed approach represents an established application of precision diagnostics in acute stroke care.

Patients with large established infarctions were historically excluded from MT because of concerns regarding limited treatment benefit and hemorrhagic complications. Recent randomized trials have challenged this exclusion, but they have not used a uniform imaging-selection strategy. RESCUE-Japan LIMIT (33) enrolled patients with ASPECTS 3–5 assessed by CT or diffusion-weighted MRI, with imaging modality varying across participants. ANGEL-ASPECT (34) enrolled patients within 24 h with anterior-circulation large-vessel occlusion and used NCCT ASPECTS 3–5 regardless of infarct-core volume, while also allowing selected patients with ASPECTS 0–2 or >5 when quantitative core volume was 70–100 mL. SELECT2 (35) enrolled patients within 24 h with internal carotid artery or M1 occlusion and defined a large ischemic core by ASPECTS 3–5 or a core volume of at least 50 mL on CT perfusion or diffusion-weighted MRI. TENSION (36) further demonstrated benefit in patients with ASPECTS 3–5 treated within 12 h, using predominantly standard-of-care imaging (with non-contrast CT as the principal modality).

Across these trials, MT improved functional outcomes in the respective enrolled populations, although the magnitude of benefit and safety profiles varied (33–37). Importantly, favorable perfusion mismatch and preserved collateral circulation were not universal inclusion requirements across the four studies. Several trials relied substantially on NCCT and ASPECTS, whereas others incorporated quantitative ischemic-core volume; therefore, advanced perfusion imaging should not be regarded as a universal prerequisite for large-core thrombectomy. The available randomized evidence supports EVT in selected patients with large established infarction, but generalizability remains dependent on the clinical population, imaging criteria, occlusion characteristics, and treatment window represented in each trial (38, 39). Trial eligibility criteria should not be interpreted as evidence that any individual imaging phenotype universally determines treatment benefit. The key characteristics and outcomes of these trials are provided in Table 2, with large-infarct and medium/distal vessel populations presented in Panels A and B, respectively.

**Table 2:** Major randomized trials informing expanded EVT selection.

Trial	Imaging selection	ASPECTS/core criteria	Occlusion site	Treatment window	Primary outcome	Treatment effect	Hemorrhagic/major safety outcome
A. Large established infarction							
RESCUE-Japan LIMIT	Primarily MRI-based assessment; CT-ASPECTS also used in some patients	ASPECTS 3–5	ICA or M1	≤6 h; 6–24 h if no early FLAIR change	mRS 0–3 at 90 days	mRS 0–3: 31.0% vs. 12.7%; RR 2.43 (95% CI 1.35–4.37)	Any ICH: 58.0% vs. 31.4%; sICH: 9.0% vs. 4.9% (RR 1.84, 95% CI 0.64–5.29)
ANGEL-ASPECT	NCCT ASPECTS; quantitative core volume used in specified ASPECTS/time-window strata	ASPECTS 3–5; or core 70–100 mL (ASPECTS 0–2; or ASPECTS >5 if 6–24 h)	Intracranial ICA and/or M1 segment	≤ 24 h	90-day ordinal mRS	Generalized OR 1.37 (95% CI 1.11–1.69)	sICH: 6.1% vs. 2.7%; any ICH 49.1% vs. 17.3%
SELECT2	NCCT ASPECTS and/or quantitative core-volume assessment by CTP or DWI-MRI	ASPECTS 3–5 or ischemic core ≥ 50 mL	ICA or M1	≤ 24 h	90-day ordinal mRS	Generalized OR 1.51 (95% CI 1.20–1.89); functional independence 20% vs. 7%, RR 2.97	sICH 1 vs. 2 patients; vascular/procedural complications occurred in EVT group; mortality similar
TENSION	Predominantly standard-of-care imaging; NCCT was the principal modality in most patients	ASPECTS 3–5	ICA and/or M1	≤ 12 h	90-day ordinal mRS	Adjusted common OR 2.58 (95% CI 1.60–4.15)	sICH 6% vs. 5%; mortality HR 0.67 (95% CI 0.46–0.98)

Trial	Vessel population	Key selection features	Treatment window	Primary outcome	Treatment effect	Major safety outcome
B. Medium/distal vessel occlusion						
ESCAPE-MeVO	Medium-vessel occlusion (M2-M3, A2-A3, P2-P3)	MeVO stroke within 12 h	≤12 h	mRS 0–1 at 90 days	41.6% vs. 43.1%; adjusted RR 0.95 (95% CI 0.79–1.15)	Mortality 13.3% vs. 8.4% (HR 1.82, 95% CI 1.06–3.12); sICH 5.4% vs. 2.2%
DISTAL	Medium or distal vessel occlusion (M2-M4, A1-A3, P1-P3)	543 patients, median age 77, median NIHSS 6	≤24 h	90-day ordinal mRS	Common OR 0.90 (95% CI 0.67–1.22; *p* = 0.50)	Mortality 15.5% vs. 14.0%; sICH 5.9% vs. 2.6% (no significant difference)
ORIENTAL-MeVO	Medium-vessel occlusion (M2, M3, A1-A3, P1-P3)	NIHSS ≥6, within 24 h, moderate-to-severe deficit	≤24 h	mRS 0–2 at 90 days	58.6% vs. 46.6%; adjusted RR 1.24 (95% CI 1.07–1.44; *p* = 0.004)	sICH more frequent with EVT (exact rates pending confirmation)

EVT, endovascular thrombectomy; ICA, internal carotid artery; M1, first segment of the middle cerebral artery; NCCT, non-contrast computed tomography; CTP, computed tomography perfusion; DWI-MRI, diffusion-weighted magnetic resonance imaging; FLAIR, fluid-attenuated inversion recovery; ASPECTS, Alberta Stroke Program Early CT Score; mRS, modified Rankin Scale; RR, relative risk; OR, odds ratio; HR, hazard ratio; ICH, intracranial hemorrhage; sICH, symptomatic intracranial hemorrhage; MeVO, medium-vessel occlusion. Treatment effects are reported according to each trial’s prespecified primary analysis and should not be interpreted as directly comparable across trials because populations, vessel definitions, imaging criteria, treatment windows, and outcome definitions differed.

### 4.2 Toward individualized selection beyond large vessel occlusion

As LVO treatment matures, attention has turned to medium vessel occlusions (MeVO) and related distal vessel populations. Observational studies have demonstrated technical feasibility and relatively high reperfusion rates with EVT, but technical success has not necessarily translated into improved functional outcomes (40, 41). In ESCAPE-MeVO (42), patients with clinically significant MeVO stroke treated within 12 h were randomized to EVT plus usual care or usual care alone; EVT did not improve the primary 90-day functional outcome, and higher mortality and symptomatic intracranial hemorrhage were observed in the EVT group. DISTAL (43) similarly found no significant improvement in 90-day functional outcome with EVT plus best medical treatment compared with best medical treatment alone across a broader medium/distal vessel population treated within 24 h. In contrast, the 2026 ORIENTAL-MeVO trial reported improved functional independence with EVT in patients with moderate-to-severe MeVO stroke, although symptomatic intracranial hemorrhage was more frequent with EVT (44).

These trials differed in vessel definitions, clinical severity, treatment windows, and imaging-selection approaches, and their results should therefore be interpreted strictly within the populations studied. Current randomized evidence does not support routine EVT for unselected MeVO populations (42, 43), while potential responder subgroups defined by occlusion location, deficit severity, collateral status, or other clinical and imaging characteristics remain important areas for investigation. MeVO should therefore be considered a distinct treatment context in which technical reperfusion success must be weighed against demonstrated functional benefit and procedural safety. The key characteristics and outcomes of these randomized trials are summarized in Table 2B. Posterior-circulation stroke, particularly basilar artery occlusion, represents another distinct clinical context with differences in patient selection, imaging assessment, and evidence base from anterior-circulation LVO.

Similarly, older adults (e.g., >80 years) and those with severe comorbidities were often underrepresented in earlier trials. Increasing clinical evidence indicates that frailty and pre-existing functional status provide critical prognostic information beyond chronological age alone for predicting outcomes after MT (10, 45–47). These measures may therefore inform individualized risk–benefit assessment, particularly in older patients or those with pre-existing disabilities, but their role as standalone treatment-selection tools remains unestablished.

## 5 Tailored reperfusion strategies

### 5.1 Bridging therapy versus direct thrombectomy: beyond a binary debate

A defining controversy in modern stroke care is whether bridging therapy (BT; intravenous thrombolysis followed by MT) provides greater clinical benefit than direct mechanical thrombectomy (dMT) in eligible patients. Randomized comparisons have primarily evaluated these strategies at the population level, whereas the proposed biological rationale for BT includes systemic thrombolysis of distal thromboemboli and potential reduction of downstream microvascular occlusion (48, 49). Conversely, dMT avoids exposure to thrombolytic agents and may reduce treatment delays in settings where intravenous thrombolysis is unlikely to provide additional benefit. These mechanisms and workflow considerations provide a rationale for ongoing investigation but do not establish phenotype-specific treatment preferences.

Multiple RCTs, including DIRECT-MT, DEVT, SWIFT-DIRECT, and DIRECT-SAFE, together with individual-participant-data and other meta-analyses, have compared BT with dMT, yielding results that support the continued evaluation of both strategies in appropriately selected patients (50–56). These studies provide population-level evidence regarding the comparative efficacy and safety of BT and dMT, while differences in treatment effects across studies or subgroups have not yet yielded a validated phenotype-based strategy for selecting one approach over the other.

Most earlier randomized trials comparing BT with direct thrombectomy predominantly used alteplase. More recently, the BRIDGE-TNK randomized trial evaluated intravenous tenecteplase (0.25 mg/kg) before thrombectomy in patients with large-vessel occlusion presenting within 4.5 h and reported a higher rate of 90-day functional independence with tenecteplase plus thrombectomy than with thrombectomy alone (52.9% vs. 44.1%; unadjusted risk ratio, 1.20; 95% CI, 1.01–1.43; *p* = 0.04) (57). Symptomatic intracranial hemorrhage occurred in 8.5% of the tenecteplase–thrombectomy group and 6.7% of the thrombectomy-alone group, and 90-day mortality was 22.3% vs. 19.9%, respectively. These findings provide contemporary randomized evidence supporting further evaluation of tenecteplase-based BT, but they do not by themselves establish a preference for tenecteplase over alteplase or define which precise patient profiles should receive BT rather than dMT.

### 5.2 Moving toward phenotype-guided reperfusion strategies

The scientific community is increasingly investigating phenotype-informed reperfusion strategies beyond a uniform “either-or” approach (58). Individual clinical, etiological, and imaging characteristics may provide prognostic or mechanistic information relevant to BT and dMT, but current evidence has not established that clot burden, collateral status, or intracranial atherosclerotic disease (ICAD) reliably modifies the relative treatment benefit of these strategies. In current practice, workflow characteristics may also influence the feasibility and timing of each approach; for example, intravenous thrombolysis may be initiated during transfer in drip-and-ship models, whereas dMT may reduce treatment delays in selected settings (59). AI-based models integrating clinical data, multimodal imaging, or molecular biomarkers may support future treatment-stratification research, while phenotype-informed reperfusion remains an area for prospective evaluation (60).

## 6 Personalized periprocedural management

### 6.1 Individualized physiological and antithrombotic management

Standardized periprocedural care protocols are increasingly being complemented by tailored physiological management. For blood pressure (BP), a uniform post-recanalization target may fail to account for individual cerebral autoregulation limits. Although autoregulation-guided BP management is physiologically plausible, randomized evidence does not currently support routine intensive BP lowering after successful EVT. The BP-TARGET trial found no reduction in intracranial hemorrhagic complications with intensive BP lowering, while ENCHANTED2/MT and OPTIMAL-BP raised concerns about intensive BP reduction and functional outcomes; BEST-II did not demonstrate a clear functional benefit from lower SBP targets (61–67). Autoregulation-guided BP management is therefore being investigated as a potential approach to individualized post-EVT physiological management.

Similarly, rigid normoglycemic targets (e.g., intensive insulin therapy) have proven ineffective and potentially harmful (68). Personalized glycemic management that avoids excessive glucose lowering and minimizes marked glycemic variability may be preferable to rigid intensive targets, although the optimal individualized strategy remains uncertain (69). Furthermore, precision targeted temperature management (maintaining 36.0–37.5 °C via automated cooling) remains an investigational neuroprotective strategy, with the potential to suppress individual inflammatory cascades triggered by reperfusion (70).

Endothelial injury post-MT necessitates precise antithrombotic therapy to balance the risks of in-situ reocclusion against sICH. For selected ICAD-related occlusions, early antiplatelet therapy may be considered, but its timing should be individualized according to intravenous thrombolysis, follow-up neuroimaging, infarct burden, hemorrhagic transformation risk, rescue angioplasty or stenting, and the procedural and vascular context (71, 72). Conversely, the optimal timing of anticoagulation for cardioembolic strokes remains complex. In the era of precision medicine, optimal antithrombotic selection may eventually incorporate pharmacogenomic information (e.g., CYP2C19 metabolizer status for clopidogrel resistance), alongside imaging-based assessments of infarct volume and BBB permeability (73).

### 6.2 Predicting secondary injury after recanalization

Emerging imaging and blood-based biomarkers may provide clinically useful prognostic information for identifying patients at higher risk of secondary injury. For example, higher postoperative hypoperfusion intensity ratio (HIR) and specific blood-based biomarkers, including eosinophil counts, have been associated with early neurological deterioration and related complications (74–77). Recent clinical evidence further supports the prognostic value of these markers, including associations between HIR and functional outcome and between eosinophil levels and hemorrhagic complications or neurological deterioration. Their current role is primarily prognostic, whereas their application to treatment selection and treatment-response prediction remains an area for further investigation (78, 79).

## 7 Future directions: AI and precision stroke systems

Addressing futile recanalization requires consideration of determinants across the stroke care pathway, from timely triage and tissue-based selection to reperfusion, periprocedural management, and subsequent functional recovery (Figure 2).

**Figure 2:**
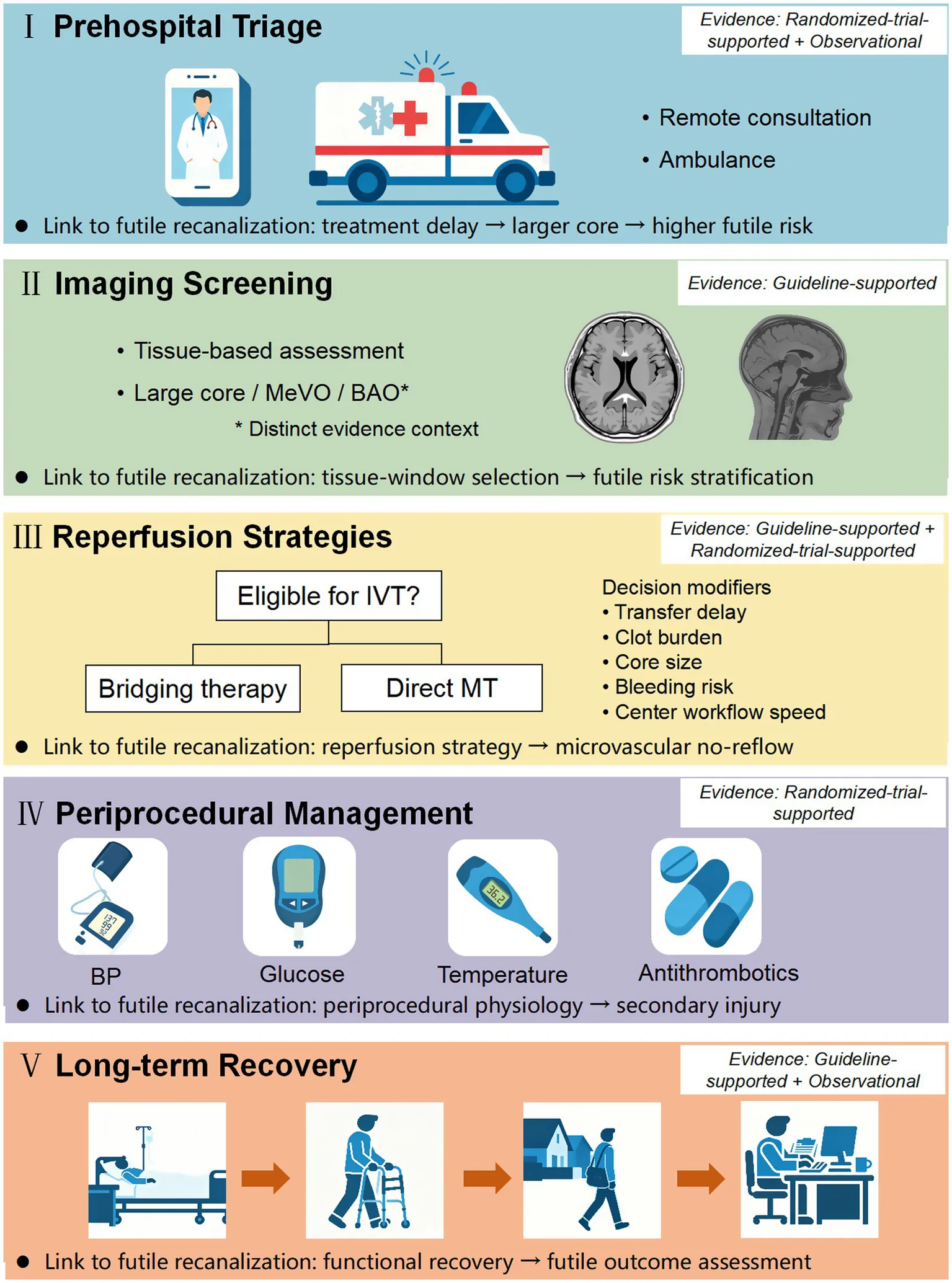
Whole-course framework of determinants and potential intervention points related to functional outcome after reperfusion. This framework illustrates how factors across the care continuum may influence the transition from successful reperfusion to functional recovery. The prehospital phase encompasses emergency triage, regional stroke networks, Mobile Stroke Units, and telestroke systems, which may facilitate timely treatment and tissue preservation. Hospital-based assessment incorporates tissue-window evaluation, infarct-core and collateral assessment, and individualized reperfusion decisions, including selected patients with large core infarction or medium/distal vessel occlusions (MeVO). Posterior-circulation presentations, including basilar artery occlusion, are considered within a distinct clinical evidence context. During the reperfusion and post-reperfusion phases, individualized treatment and periprocedural management may influence tissue injury and functional outcome. Early rehabilitation and long-term follow-up extend the care continuum toward sustained functional recovery. Components are labeled as guideline-supported, randomized-trial-supported, observational, or preclinical.

### 7.1 AI-enabled precision decision support

System-level approaches may reduce treatment delays that can contribute to poor outcomes after reperfusion. Regional stroke protocols and Mobile Stroke Units (MSUs) equipped with CT scanners may facilitate earlier recognition, triage, and transfer of patients with suspected LVO to EVT-capable centers (80–84). Integrated telestroke networks may facilitate timely remote assessment and access to reperfusion pathways (85, 86). These system-level approaches primarily influence treatment timing and tissue preservation, providing an indirect pathway through which stroke systems may affect outcomes after reperfusion.

Artificial intelligence (AI) may support precision stroke care by assisting with automated image interpretation and multimodal prognostic stratification. Deep learning models applied to non-contrast CT and multimodal MRI have been developed to assist with large-vessel occlusion detection, ischemic core estimation, and collateral assessment (87, 88). Beyond imaging, AI pipelines are being investigated to integrate clinical baseline data, laboratory parameters, and multi-omics information for prediction of recanalization success, hemorrhagic risk, and long-term functional outcomes (60). These applications are currently centered on diagnostic and prognostic support, with ongoing efforts to establish their generalizability, calibration, and clinical impact across diverse populations and care settings. Importantly, prediction of clinical outcomes should not be equated with prediction of treatment benefit; the latter requires evidence of treatment-effect modification and prospective clinical validation.

### 7.2 Targeting microcirculatory protection: the next frontier

Because large-vessel recanalization does not necessarily equate to tissue reperfusion, microcirculatory protection represents an important frontier for precision-oriented stroke research. Experimental approaches targeting immunomodulatory pathways, including neutrophil extracellular traps (NETs) and inflammatory mediators such as lipocalin 2 (LCN2), have been proposed as potential strategies to modify thrombo-inflammatory injury (89–92). These findings provide a translational basis for investigating targeted immunomodulation and related individualized pharmacological strategies as adjuncts to reperfusion therapy (Figure 3).

**Figure 3:**
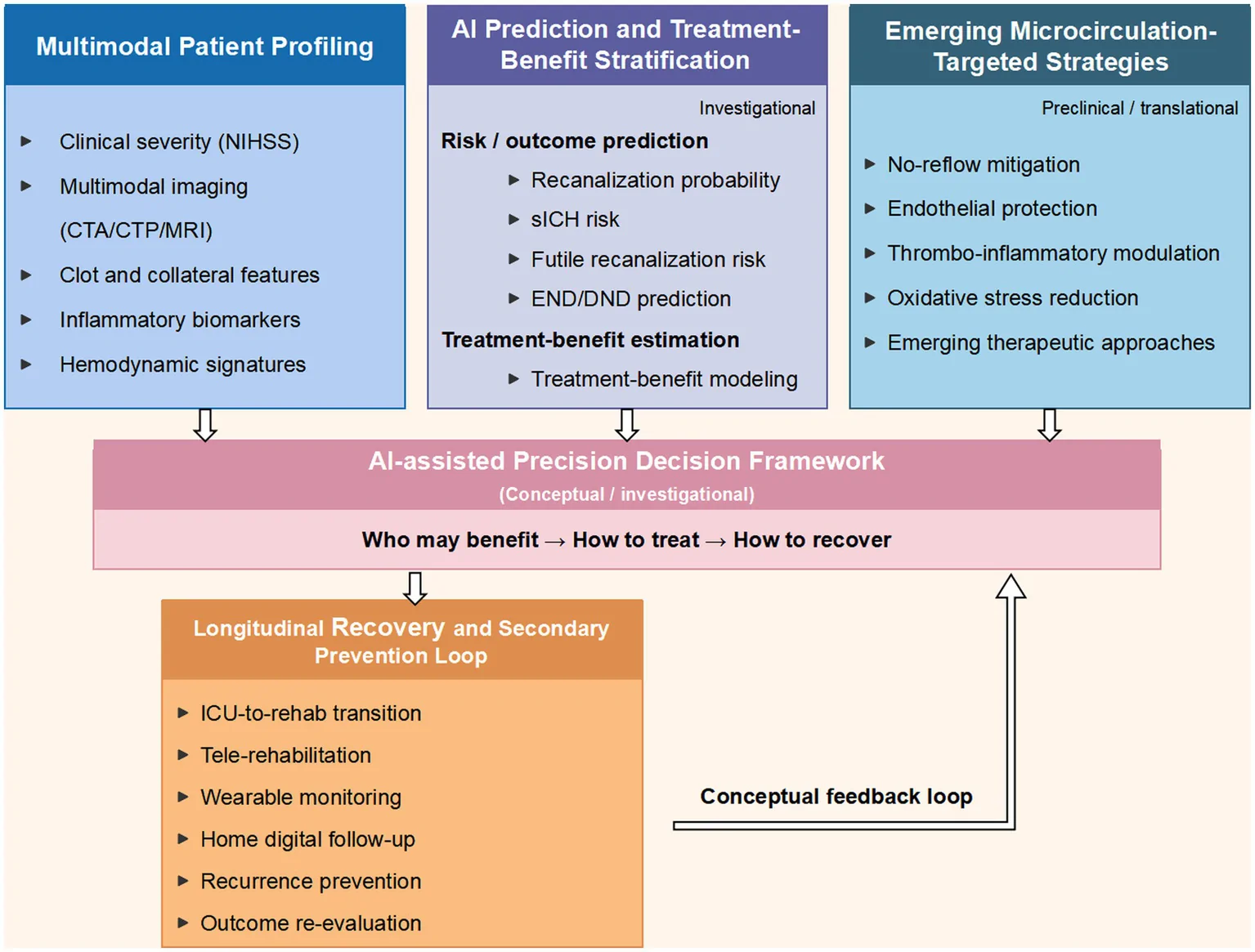
AI-driven precision stroke ecosystem across the full care continuum. This schematic illustrates a conceptual framework for future post-thrombectomy stroke management. Upstream modules consisting of multimodal patient phenotyping, AI-guided prognostic stratification, and preclinically investigated microcirculation-targeted immunotherapy converge within a proposed AI-assisted decision-support framework to inform (but not currently determine) reperfusion-related treatment and rehabilitation strategies. Downstream modules include intensive care monitoring, rehabilitation, digital home follow-up, and long-term secondary prevention, with outcome reassessment providing a feedback pathway for subsequent decision adjustment. This figure represents a conceptual framework for future development; AI components are not currently validated for routine clinical use. Established care, randomized-trial-supported approaches, observational evidence, and preclinical concepts are visually distinguished.

## 8 Discussion

Despite the remarkable clinical advances achieved with MT, the persistent occurrence of futile recanalization highlights several unresolved controversies and translational gaps. Foremost among these, a key unresolved question is whether BT or dMT may confer differential benefit across clinically meaningful patient phenotypes. No prospectively validated biomarker or phenotype currently identifies patients who are more likely to benefit from one strategy over the other. Future trials incorporating predefined biological, imaging, or clinical subgroups may help determine whether treatment-effect heterogeneity exists and whether such information can support individualized reperfusion strategies.

Furthermore, a significant research gap exists between our macroscopic imaging capabilities and microscopic tissue realities. While interventionalists can successfully reopen large vessels, the field currently lacks widely validated, real-time clinical tools to assess microvascular “no-reflow” within the angiography suite. Additionally, although preclinical studies have identified molecular drivers of reperfusion injury, including NETs and ROS, validated blood-based biomarkers and rapid molecular tools for guiding individualized neuroprotective strategies remain unavailable in the hyperacute setting.

Beyond biological and technical challenges, translating these precision innovations into widespread clinical practice faces substantial implementation barriers that may threaten equitable care. The development of robust AI algorithms is frequently hindered by insufficiently diverse datasets, raising concerns regarding generalizability and algorithmic bias when applied to underrepresented populations. Moreover, precision diagnostics, including advanced imaging workflows, multi-omics profiling, and specialized stroke systems such as Mobile Stroke Units, may require substantial financial and infrastructural investment. If these technologies remain concentrated in well-resourced comprehensive stroke centers, the transition toward personalized medicine could inadvertently widen global health disparities. Addressing cost-effectiveness, accessibility, and equitable implementation should therefore remain central priorities for future stroke policy.

Several limitations should be considered when interpreting this review. First, as a narrative review, the literature selection and synthesis may be subject to selection and interpretive bias despite the use of a structured search strategy and prioritization of randomized trials, meta-analyses, guidelines, and large observational studies. Second, the definition of futile recanalization varies across studies according to reperfusion thresholds, mTICI/eTICI grades, functional outcome cutoffs, assessment time points, and baseline or pre-stroke disability, limiting direct comparison of reported estimates and associations. Third, evidence supporting many proposed precision-medicine approaches remains observational, mechanistic, or preclinical. Prognostic associations identified by imaging or circulating biomarkers should not be interpreted as evidence of treatment-effect modification, and most candidate biomarkers have not been validated for treatment selection. Similarly, AI-based approaches remain limited by issues of external validation, calibration, dataset shift, bias, and prospective clinical impact. Finally, whether phenotype-guided reperfusion or microcirculation-targeted interventions improve patient outcomes remains uncertain and requires prospective studies designed to evaluate treatment-effect heterogeneity and clinical benefit.

Ultimately, supported by landmark trials, MT has fundamentally improved outcomes across a broad spectrum of stroke populations; however, angiographic success represents only the beginning rather than the endpoint of neurological recovery. The next phase of stroke care may require moving beyond reliance on angiographic reperfusion endpoints toward a personalized medicine paradigm. At the systemic level, AI-assisted triage may facilitate individualized prehospital routing. At the diagnostic level, molecular profiling and multimodal imaging may complement conventional time-based selection with precision tissue evaluation. At the therapeutic level, patient-specific reperfusion algorithms and targeted neuroprotective strategies may provide future opportunities to mitigate microcirculatory failure. Only by integrating precision medicine with equitable implementation can we bridge the gap between angiographic success and meaningful functional recovery in cerebrovascular disease.
