# Supplementary material for: Precision medicine for futile recanalization after mechanical thrombectomy in acute ischemic stroke: mechanisms, diagnostics, and tailored therapies
Source: Front Neurol. 2026 Oct 8;17:1962902. doi: 10.3389/fneur.2026.1962902 (PMC13689838; doi:10.3389/fneur.2026.1962902)
Supplement: Supplementary file 1 [file Data_Sheet_1.pdf]

Supplementary Table 1. Structured literature search strategy and review scope

Item	Description
Review type	Narrative review informed by a structured literature search.
Databases searched	PubMed, Web of Science, and Embase
Final search date	12 Sep 2026
Language restriction	English-language peer-reviewed publications.
Core acute ischemic stroke terms	“acute ischemic stroke” OR “acute ischaemic stroke” OR “ischemic stroke” OR “ischaemic stroke”
Core mechanical thrombectomy terms	“mechanical thrombectomy” OR “endovascular thrombectomy” OR “endovascular treatment” OR “endovascular therapy” OR EVT
Core futile recanalization terms	“futile recanalization” OR “futile recanalisation” OR “futile reperfusion” OR “poor outcome despite successful recanalization” OR “poor outcome despite successful reperfusion”
Targeted mechanistic and microcirculatory searches	Targeted searches using terms such as “no-reflow,” “no reflow,” “microvascular dysfunction,” “microcirculatory dysfunction,” “persistent hypoperfusion,” “tissue-level hypoperfusion,” “endothelial injury,” “thrombo-inflammation,” “neutrophil extracellular traps,” NETs, “reactive oxygen species,” ROS, “blood – brain barrier,” pericyte, “lipocalin 2,” and LCN2 were conducted to support the mechanistic and microcirculatory sections.
Targeted clinical strategy and periprocedural searches	Targeted searches using terms such as “large core,” “large infarct,” “large ischemic core,” ASPECTS, “large vessel occlusion,” LVO, “medium vessel occlusion,” MeVO, “distal vessel occlusion,” “posterior circulation stroke,” “basilar artery occlusion,” “bridging therapy,” “bridging thrombolysis,” “direct thrombectomy,” alteplase, tenecteplase, “blood pressure,” autoregulation, antithrombotic, antiplatelet, and anticoagulation were conducted to support the reperfusion-strategy and periprocedural management sections.
Targeted precision diagnostics, biomarkers, and AI searches	Additional targeted searches using terms such as biomarker, “hypoperfusion intensity ratio,” HIR, eosinophil, “artificial intelligence,” “machine learning,” “deep learning,” “multi-omics,” pharmacogenomics, CYP2C19, and imaging were conducted to support the precision diagnostics, biomarker, and AI sections.
Search approach	A broad-to-specific approach was used, beginning with a core search of acute ischemic stroke, mechanical thrombectomy, and futile recanalization terms, followed by topic-specific supplementary searches. Search combinations were adapted to the topic and database syntax.
Representative search strategy	Core search: ("acute ischemic stroke" OR "acute ischaemic stroke" OR "ischemic stroke" OR "ischaemic stroke") AND ("mechanical thrombectomy" OR "endovascular thrombectomy" OR "endovascular treatment" OR "endovascular therapy" OR EVT) AND ("futile recanalization" OR "futile recanalisation" OR "futile reperfusion" OR "poor outcome despite successful recanalization" OR "poor outcome despite successful reperfusion")<br>This core search was supplemented by targeted PubMed searches combining relevant acute ischemic stroke and mechanical thrombectomy concepts with topic-specific terms addressing mechanistic and microcirculatory processes, clinical and reperfusion strategies, periprocedural management, precision diagnostics, biomarkers, imaging, and artificial intelligence.
Inclusion criteria	Peer-reviewed studies and formal clinical guidelines or consensus documents addressing acute ischemic stroke in the context of endovascular therapy/mechanical thrombectomy, particularly those examining futile recanalization, functional outcomes after successful reperfusion, mechanisms of reperfusion failure, patient selection, reperfusion strategies, periprocedural management, biomarkers, imaging, or artificial intelligence. RCTs, systematic reviews/meta-analyses, formal clinical guidelines or consensus documents, and large observational studies were prioritized. Preclinical studies were selectively included when providing mechanistic evidence relevant to futile recanalization or microvascular injury.
Exclusion criteria	Case reports, small case series (<10 patients), studies focused on non-ischemic cerebrovascular diseases, and publications lacking clinically or mechanistically relevant outcomes were excluded.
Handling of overlapping cohorts	When multiple publications appeared to derive from overlapping cohorts or trials, they were not automatically excluded if they addressed distinct outcomes or research questions. Related reports were interpreted as potentially non-independent evidence, and the most comprehensive or directly relevant report was prioritized when analyses substantially overlapped.
Evidence synthesis approach	Narrative synthesis emphasizing study design, population characteristics, reperfusion and functional outcome definitions, imaging and biomarker measures, treatment effects, and methodological limitations. Clinical interpretation prioritized RCTs, meta-analyses, and contemporary guidelines over observational and preclinical evidence; preclinical findings were considered hypothesis-generating. Conflicting evidence was assessed according to study quality, directness, and consistency, with uncertainty and heterogeneity acknowledged.

Note. AI, artificial intelligence; ASPECTS, Alberta Stroke Program Early CT Score; EVT, endovascular treatment; HIR, hypoperfusion intensity ratio; LCN2, lipocalin 2; LVO, large vessel occlusion; MeVO, medium vessel occlusion; NETs, neutrophil extracellular traps; RCTs, randomized controlled trials; ROS, reactive oxygen species.
